# Stress-Induced Enhanced Long-Term Potentiation and Reduced Threshold for N-Methyl-D-Aspartate Receptor- and β-Adrenergic Receptor-Mediated Synaptic Plasticity in Rodent Ventral Subiculum

**DOI:** 10.3389/fnmol.2021.658465

**Published:** 2021-04-22

**Authors:** Julia C. Bartsch, Monique von Cramon, David Gruber, Uwe Heinemann, Joachim Behr

**Affiliations:** ^1^Department of Psychiatry and Psychotherapy, Charité-Universitätsmedizin Berlin, Berlin, Germany; ^2^Department of Psychiatry, Psychotherapy and Psychosomatic Medicine, Brandenburg Medical School, Neuruppin, Germany; ^3^Institute of Neurophysiology, Charité-Universitätsmedizin Berlin, Berlin, Germany; ^4^Faculty of Health Sciences Brandenburg, Joint Faculty of the University of Potsdam, Brandenburg University of Technology Cottbus-Senftenberg and Brandenburg Medical School, Potsdam, Germany

**Keywords:** subiculum, long-term potentiation, stress, norepinephrine, metaplasticity, β-adrenergic receptor, hippocampus

## Abstract

Stress is a biologically relevant signal and can modulate hippocampal synaptic plasticity. The subiculum is the major output station of the hippocampus and serves as a critical hub in the stress response network. However, stress-associated synaptic plasticity in the ventral subiculum has not been adequately addressed. Therefore, we investigated the impact of a single exposure to an inherently stressful two-way active avoidance conditioning on the induction of long-term potentiation (LTP) at CA1—subiculum synapses in ventral hippocampal slices from young adult rats 1 day after stressor exposure. We found that acute stress enhanced LTP and lowered the induction threshold for a late-onset LTP at excitatory CA1 to subicular burst-spiking neuron synapses. This late-onset LTP was dependent on the activation of β-adrenergic and glutamatergic *N*-methyl-D-aspartate receptors and independent of D1/D5 dopamine receptor activation. Thereby, we present a cellular mechanism that might contribute to behavioral stress adaptation after acute stressor exposure.

## Introduction

When faced with challenging environmental situations (stressors), it is crucial for an individual to rapidly adapt neuronal activity underlying cognition and behavior to cope with these challenges. Therefore, the brain has evolved a complex stress-response system (Joëls and Baram, 2009). The hypothalamic—pituitary—adrenal axis and the locus coeruleus—norepinephrine system are the two main brain networks that are systematically associated with stress. Research in rodents and humans has shown that the hippocampus, a medial temporal lobe structure crucially implicated in memory formation, is highly susceptible to stress (reviewed in Kim et al., 2006). Acute stress has been shown to alter hippocampal synaptic plasticity (reviewed in Howland and Wang, 2008; and Kim et al., 2015) including long-term potentiation (LTP), the activity-dependent increase in synaptic efficacy, which is considered a cellular model of learning and memory (Bliss and Lømo, 1973; Martin et al., 2000; Malenka and Bear, 2004). Studies by MacDougall and Howland (2013a,b) have demonstrated that acute stress disrupts LTP in rat dorsal subiculum. While the dorsal hippocampus is especially associated with spatial learning, the ventral hippocampus is involved in context-dependent processes (Jarrard, 1995; Moser and Moser, 1998; Maren, 1999; Sharp, 1999; Fanselow, 2000; Bannerman et al., 2004; Segal et al., 2010). The subiculum serves as the major output node of the hippocampal formation (O’Mara et al., 2000; Aggleton and Christiansen, 2015). Particularly, the ventral subiculum integrates and orchestrates the stress response due to its feedback control of the hypothalamic—pituitary—adrenal axis and its dense norepinephrine innervation (Oleskevich et al., 1989; Herman et al., 1998; Mueller et al., 2004; O’Mara, 2005; Herman and Mueller, 2006). Norepinephrine acts *via* G-protein-coupled adrenergic receptors (AR), initiating intracellular signaling cascades (Chay et al., 2016). Hippocampal pyramidal cells express a high density of β-ARs and activation of β-ARs can potently facilitate hippocampal LTP (Duncan et al., 1991; Nicholas et al., 1993; Hillman et al., 2005; Guo and Li, 2007; O’Dell et al., 2010). Two-way active avoidance conditioning is a higher-order operant learning task known to rely on proper function of amygdala (Savonenko et al., 2003), hippocampal structures (Schwegler et al., 1981; Becker et al., 1997) and basal forebrain regions (Miyamoto et al., 1985). It poses a stressful but controllable condition for rats (Tsoory et al., 2007) and has been previously used for the analysis of anxiety- and stress-mediated behavior in adult life (Tsoory et al., 2007; Gruber et al., 2015). Previous studies have shown that learning and performance in this task critically depends on regulation of stress-response mechanisms (Brush, 2003; Asai et al., 2004; Kademian et al., 2005).

Given the well-known susceptibility of hippocampal synaptic plasticity to stress and the paucity of findings related to the subiculum, the main hippocampal output node and prominent stress integrator, we investigated the impact of acute stress on the induction of LTP in the subiculum in rodent ventral hippocampal slices. Our results show that a single, stressful two-way active avoidance conditioning during adulthood results in an enhanced LTP and a reduced threshold for a β-AR- and *N*-methyl-D-aspartate receptor (NMDAR)-dependent LTP at glutamatergic CA1 to subicular burst-spiking neuron synapses. These cellular mechanisms might contribute to the behavioral adaptations after stressor exposure.

## Materials and Methods

### Study Design

**Figure 1:**
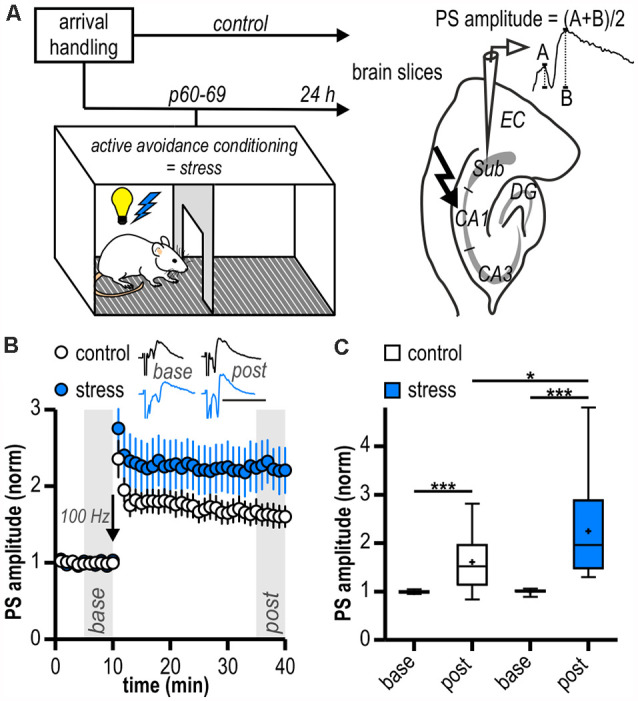
Long-term potentiation (LTP) at ventral hippocampal CA1—subiculum synapses is enhanced following acute stress in adulthood. **(A)** Study design. Schematic drawing on the right illustrates the placement of the stimulation and recording electrodes in the hippocampal slice and the calculation of the population spike (PS) amplitude. **(B)** Time course of mean normalized PS amplitudes at CA1—subiculum synapses. LTP in the subicular field following high-frequency stimulation (HFS, 100 Hz, arrow) is significantly enhanced in stressed rats. Inset shows sample PS traces before (*base*, min 6–10) and after (*post*, min 36–40) HFS, scale bars: amplitude normalized to baseline of control, 50 ms. **(C)** Quantification of mean normalized PS amplitudes (control: *n* = 15/10, paired Student’s *t*-test, ***p < 0.001; stress: *n* = 12/6, Wilcoxon signed ranked test, ***p < 0.001; control vs. stress, unpaired Student’s *t*-test, *p < 0.05).

All procedures were approved by the local health authority (Landesamt für Gesundheit und Soziales Berlin) and adhered to national and international guidelines (directive 2010/63/EU of the European Parliament and of the council of 22 September 2010 for animal experiments). Male Wistar rats (Harlan or Forschungseinrichtungen für Experimentelle Medizin Charité, Germany; Janvier, France) were kept in groups of 2–4 per cage. To avoid possible interactions with stress hormone levels and the stress response due to the fluctuations of hormone levels associated with the estrus cycle of female rats, only male rats were used in this study. Rats were delivered on postnatal day (p) 21–60, after weaning and were randomly assigned to either the control group or adult stress group. Control rats were not exposed to any adverse events. Adult stress rats were subjected to an adult stress protocol by a single two-way shuttle box training session once between p 60 and p 69. This protocol was validated for the analysis of anxiety-mediated behavior (Fernández-Teruel et al., 1991). It poses a stressful but controllable condition for rats (Tsoory et al., 2007) and has been previously used for the analysis of anxiety- and stress-mediated behavior in adult life (Tsoory et al., 2007; Gruber et al., 2015). The training session routinely contained 100 trials with inter-trial intervals of 60 ± 12 s. Three animals were exposed to only 40 trials and were included in the analysis since excluding these rats from the analysis did not significantly change results. Each trial consisted of a 10 s tone with the presentation of a light signal, immediately followed by an electric foot shock (0.8 mA, 10 s), if not prevented by shuttling to the other compartment of the shuttle box during the signal (termed avoidance shuttle). Animals’ performance was analyzed as number of avoidance shuttles/number of trials. All *in vivo* experiments were performed during the light phase. One day after stress exposure, *ex vivo* electrophysiology was performed (Figure 1A).

### Slice Preparation

Twenty-four hours after stress exposure, rats were decapitated under deep anesthesia (nitrous oxide/isoflurane) and the brains were quickly removed. Horizontal slices (400 μm) containing the ventral pole of the hippocampal formation and the entorhinal cortex were obtained with a Leica VT1200S vibratome (Leica Microsystems CMS, Mannheim, Germany). The preparation was performed in ice-cold, oxygenated (95% O_2_, 5% CO_2_) artificial cerebrospinal fluid (ACSF) and the slices were transferred for storage to an interface recording chamber continuously perfused (1.5–2 ml/min) with oxygenated and prewarmed (34°C) ACSF. The composition of the ACSF was as follows (in mM): NaCl 129, Na_2_PO_4_ 1.25, NaHCO_3_ 26, KCl 3, CaCl_2_ 1.6, MgSO_4_ 1.8, glucose 10 at a pH of 7.4.

### Electrophysiology

Electrophysiological recordings were performed at near physiological temperatures (32–34°C) with a SEC10LX amplifier (NPI Electronic, Tamm, Germany). Signals were low-pass filtered at 3 kHz, sampled at 10 kHz by an ITC-16 interface (Instrutech Corp., Great Neck, NY, USA) and processed by TIDA software (HEKA GmbH, Lambrecht, Germany). Field potentials were recorded with glass pipettes containing ACSF placed in the middle portion of the subiculum. Single-cell recordings in bursting pyramidal cells of the subiculum were performed with sharp microelectrodes (40–100 MΩ) filled with 2.5 M potassium acetate in current-clamp bridge mode at resting membrane potential. When recording from single subicular neurons, we focused on bursting pyramidal cells as they constitute the majority of subicular neurons (60–100% depending on the proximodistal subregion; O’Mara et al., 2001; Menendez de la Prida et al., 2002). To study synaptic plasticity at glutamatergic synapses, all experiments at the single-cell level were performed in the presence of bicuculline (5 μM) to block GABA_A_ receptor-mediated responses. To prevent polysynaptic responses, concentrations of MgSO_4_ and CaCl_2_ were elevated to 4 mm each. Population spikes (PS) or excitatory postsynaptic potentials (EPSP) were evoked at 0.1 Hz by stimulation (100 μs) of CA1 efferents with an ACSF-filled patch pipette or a bipolar stimulating electrode. In single-cell recordings, input-output curves were conducted by stimulating CA1 efferents with increasing stimulation intensities (10 steps at 0.1 Hz between minimal stimulation strength needed to elicit an EPSP and maximal stimulation strength without action potential firing). Response amplitudes were averaged from three consecutive pulses at a given stimulation intensity. Baseline responses were recorded at 0.1 Hz for at least 10 min and the stimulus intensity was set to evoke amplitudes of 30–50% of the maximum response. Depending on the rationale of the experiment, different high-frequency stimulation (HFS) protocols were used for the induction of LTP. In local field potential recordings, 4 × 100 pulses at 100 Hz with 10 s inter-train intervals, and the same HFS protocol or 10 pulses at 40 Hz in sharp microelectrode recordings. Changes in synaptic strength were measured for a period of 30 min after induction. Amplitudes of evoked PS or EPSP were normalized to baseline values. Calculation of PS amplitudes is illustrated in Figure 1A. LTP was calculated by averaging the responses collected during the last 5 min of each experiment. Data points were binned from six consecutive responses. Paired-pulse index (PPI) was investigated by analyzing the ratio of the second to the first synaptic response (EPSP2/EPSP1) at an inter-stimulus interval of 60 ms. The coefficient of variation (CV) was calculated as CV^2^ = (SD of EPSP/mean of EPSP)^2^ from a stable 2 min long recording period of baseline and LTP, respectively.

### Drugs

The following drugs were used: (-)-bicuculline methiodide ((*R*-(R*,S*))-5-(6,8-dihydro-8-oxofuro(3,4-e)-1,3-benzodioxol-6-yl)-5,6,7,8-tetrahydro-6,6-dimethyl-1,3-dioxolo(4,5-g)isoquinolinium iodide), 5 μM; SCH23390 hydrochloride ((R)-(+)-7-chloro-8-hydroxy-3-methyl-1-phenyl-2,3,4,5-tetra-hydro-1H-3-benzazepine hydrochloride), 10 μM; D-AP5 (D-(-)-2-amino-5-phosphonopentanoic acid), 100 μM; propranolol, 2 μM.

### Data Analysis/Statistics

Electrophysiological data were analyzed offline with Clampfit software (Molecular Devices Corporation, Sunnyvale, CA, USA), TIDA software (HEKA GmbH, Lambrecht, Germany) and GraphPad Prism (GraphPad Software, La Jolla, CA, USA). Comparisons between two groups were analyzed by appropriate *t*-tests or nonparametric equivalents. Multi-group comparisons were analyzed with one-way analysis of variance (ANOVA) followed by Dunnett’s *post hoc* test against the stress group, or mixed model two-way ANOVA with time and group as factors followed by *post hoc* Bonferroni’s multiple comparison test. Correlation between LTP magnitude and rats’ performance during the behavioral task was assessed with Pearson correlation coefficient. Only for correlation analysis, LTP was averaged for a rat when more than one slice/cell per rat was recorded. Statistical significance level was set to *p* < 0.05. Values are given as mean ± standard error of the mean (SEM) or box and whisker plots (box: 25th to 75th percentiles, whiskers 5th and 95th percentiles). Numbers given in text (x/y) refer to numbers of slices or neurons (x) recorded from different animals (y).

## Results

### Enhanced Subicular LTP Following Acute Stress

In local field potential recordings in the subiculum, there were no differences in mean magnitudes of baseline amplitudes between groups (control: 0.49 ± 0.05 mV, *n* = 15/10; stress: 0.43 ± 0.06 mV, *n* = 12/6, *p* = 0.41). Stable LTP could be readily induced in both experimental groups (control: 1.61 ± 0.14, *n* = 15/10, *p* < 0.001; stress: 2.25 ± 0.29, *n* = 12/6, *p* < 0.001; Figures 1B,C). However, LTP in slices from the stress group was significantly larger than in control experiments (*p* < 0.05; Figure 1C).

Subicular pyramidal neurons can be classified into regular-spiking and burst-spiking neurons, and both show cell type-specific LTP (Wozny et al., 2008a,b). Depending on the proximodistal axis, burst-spiking neurons are the dominating cell type (60–100%; O’Mara et al., 2001; Menendez de la Prida et al., 2002). To shed light on the underlying mechanisms of the stress-enhanced subicular LTP, we therefore turned to the single-cell level and performed sharp microelectrode recordings at excitatory CA1—subiculum synapses in burst-spiking neurons.

**Figure 2:**
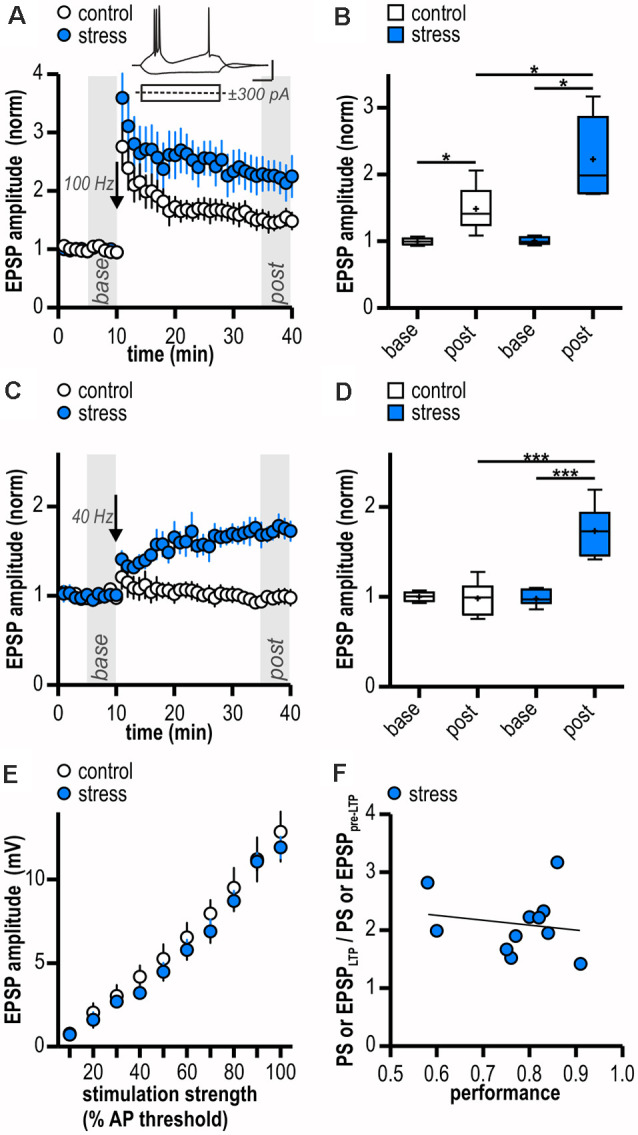
Facilitated excitatory LTP in ventral subicular burst-spiking neurons following acute stress in adulthood. **(A)** Time course of mean normalized excitatory post synaptic potentials (EPSP) amplitudes at CA1—subiculum synapses to burst-spiking pyramidal neurons. Inset shows sample voltage responses of a subicular bursting neuron to depolarizing (+300 pA) and hyperpolarizing (−300 pA) current pulses. LTP at glutamatergic CA1—subiculum burst-spiking synapses following HFS (arrow, 100 Hz) is enhanced in stressed rats. **(B)** Quantification of mean normalized EPSP amplitudes (control: *n* = 6/4, paired Student’s *t*-test, *p < 0.05; stress: *n* = 5/4, paired Student’s *t*-test, *p < 0.05; control vs. stress, unpaired Student’s *t*-test, *p < 0.05). **(C)** Attenuated HFS (arrow, 10 pulses at 40 Hz) induces LTP at glutamatergic CA1—subiculum synapses selectively in stressed rats. **(D)** Quantification of mean normalized EPSP amplitudes (control: *n* = 6/5, paired Student’s *t*-test, *p* = 0.83; stress: *n* = 8/8, paired Student’s *t*-test, ***p < 0.001; control vs. stress, unpaired Student’s *t*-test, ***p < 0.001). **(E)** Stress experience does not alter basal excitatory synaptic transmission at glutamatergic CA1—subiculum burst-spiking synapses (both *n* = 6/5; mixed model two-way ANOVA: stimulation strength: *F*_(9,90)_ = 118.04, *p* < 0.0001, group: *F*_(1,90)_ = 0.51, *p* = 0.49, interaction: *F*_(9,90)_ = 0.24, *p = 0.99*). **(F)** Magnitude of LTP and performance of rats in the active avoidance conditioning test do not correlate (Pearson correlation: *r*_(9)_ = -0.17, *p* = 0.63).

In line with our results from field potential recordings, LTP at glutamatergic CA1—subiculum burst-spiking synapses could be readily induced in both experimental groups (control: 1.49 ± 0.14, *n* = 6/4, *p* < 0.05; stress: 2.23 ± 0.28, *n* = 5/4, *p* < 0.05; Figures 2A,B) and was enhanced in stressed animals (*p* < 0.05; Figure 2B). To test for an altered induction threshold for LTP that can be masked by strong HFS, we dampened the stimulation protocol. An attenuated HFS protocol (10 pulses at 40 Hz) failed to induce plasticity in control rats (0.98 ± 0.08, *n* = 6/5, *p* = 0.83; Figures 2C,D). In stressed rats, however, it resulted in a robust late-onset LTP (1.73 ± 0.10, *n* = 8/8, *p* < 0.001; Figures 2C,D). The time course of this LTP is reminiscent of a previously reported form of synaptic plasticity at CA1—subiculum burst-spiking synapses (Bartsch et al., 2015). Hence, stressed rats show a reduced threshold for LTP in subicular burst-spiking neurons.

To determine if synaptic transmission in stressed animals is already altered prior to LTP induction, we compared basal stimulus-induced synaptic transmission between control animals (handling only) and stressed animals (24 h after two-way active avoidance conditioning). A stimulation electrode was placed to stimulate CA1 efferents and evoke EPSPs in individual subicular bursting neurons. Amplitudes of evoked EPSPs increased with increasing stimulation strength and no significant differences were observed between control and stressed rats (control: from 0.77 ± 0.26 mV to 12.84 ± 1.78 mV; stress: from 0.71 ± 0.21 mV to 11.90 ± 0.65 mV; both *n* = 6/5; mixed model two-way ANOVA: stimulation strength: *F*_(9,90)_ = 118.04, *p* < 0.0001, group: *F*_(1,90)_ = 0.51, *p* = 0.49, interaction: *F*_(9,90)_ = 0.24, *p* = 0.99; Figure 2E). We conclude that stress experience does not alter basal excitatory synaptic transmission at glutamatergic CA1—subiculum burst-spiking synapses.

Besides the stressful component, two-way active avoidance conditioning also comprises a learning component which might influence hippocampal plasticity. However, we found no correlation between LTP magnitude and rats’ performance during the task (Pearson correlation: *r*_(9)_ = −0.17, *p* = 0.63; Figure 2F) suggesting that LTP facilitation is indeed due to stress rather than learning during the behavioral paradigm.

### Stress-Induced Late-Onset LTP Comprises Pre- and Postsynaptic Mechanisms

**Figure 3:**
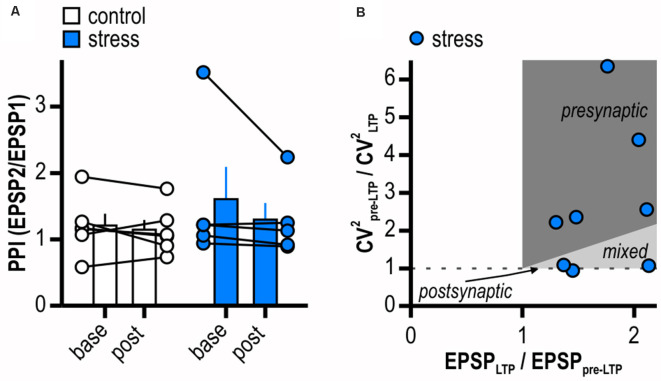
LTP in stressed rats is expressed pre- and postsynaptically. **(A)** Quantification of paired-pulse index (PPI). Circles depict the mean value of single neurons before (*base*) and after (*post*) LTP induction, columns represent mean ± SEM of all recorded neurons. Paired-pulse index (PPI) does not change after induction of LTP in stressed rats (control: *n* = 6/5, paired Student’s *t*-test, *p* = 0.50; stress: *n* = 5/5, paired Student’s *t*-test, *p* = 0.28). **(B)** Plot relating the ratio of the coefficients of variation (CV) squared to the ratio of the mean EPSP amplitudes in stressed rats. Classically, for a single cell the locus for potentiation is postsynaptic when data is on horizontal line, presynaptic when above the identity line or both when between the horizontal and the identity line.

We used different approaches to determine the expression site of this late-onset LTP in stressed rats. PPI and CV^2^ were calculated from experiments shown in Figure 2C. The PPI investigates the ability of synapses to increase transmitter release upon the second of two closely spaced afferent stimuli and depends on residual Ca^2+^ levels in the presynaptic terminal (Zucker and Regehr, 2002). Notably, in two recordings of the stress group no paired-pulse protocol was applied and one outlier displaying a baseline PPI of 6.31 was removed from analysis (Grubbs’ test). LTP induction in stressed animals did not significantly change PPI (from 1.61 ± 0.49 to 1.30 ± 0.25, *n* = 5/5, *p* = 0.28; Figure 3A) giving no evidence for an increase in transmitter release dependent on altered intraterminal Ca^2+^ concentrations. A change in CV^2^ that accompanies alterations in synaptic efficacy is likewise used to differentiate between pre- and postsynaptic mechanisms (Faber and Korn, 1991). Supporting a presynaptic mechanism, on average, LTP induction went along with a change in CV^2^ (baseline: 0.05 ± 0.01, LTP: 0.03 ± 0.01, *n* = 8/8, paired Student’s *t*-test, *p* < 0.05). However, plotting the ratio of CV^2^ before and after LTP induction to the ratio of the mean EPSP amplitudes after and prior to LTP induction in stressed rats revealed five out of eight cells with a presynaptic locus for potentiation and three out of eight cells with no evidence for increased transmitter release after LTP induction (Figure 3B). Taken together, our results therefore support contributing presynaptic expression mechanisms while additional postsynaptic ones cannot be excluded.

### Stress-Induced Late-Onset LTP Depends on the Activation of NMDAR and β-AR but Is Independent From D1/D5R

**Figure 4:**
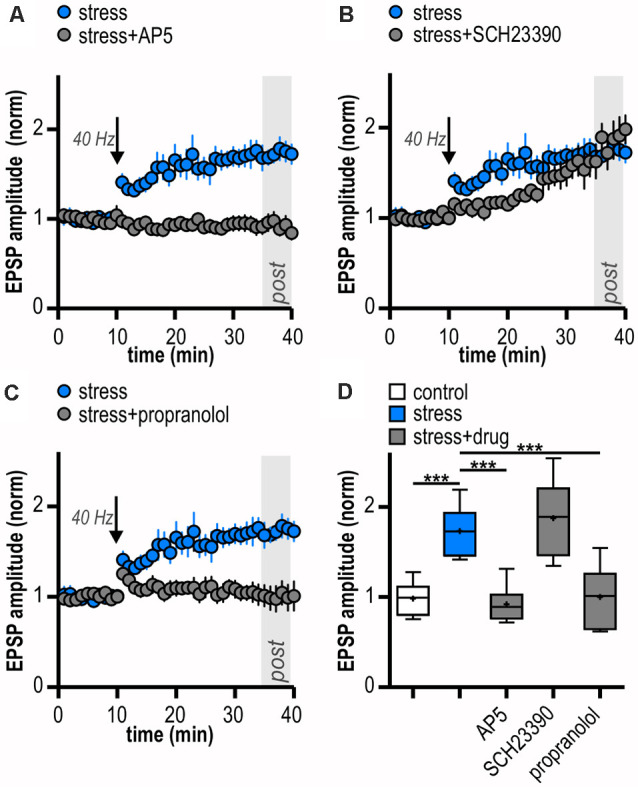
LTP in stressed rats is induced by activation of *N*-methyl-D-aspartate receptors (NMDAR) and β-adrenergic receptors (β-AR). **(A)** Bath-application of the NMDAR antagonist AP5 blocks LTP in stressed rats. **(B)** The D1/D5R antagonist SCH23390 does not block the induction of LTP in stressed rats. **(C)** The β-AR antagonist propranolol prevents the induction of LTP in stressed rats. **(D)** Quantification of mean normalized EPSP amplitudes following attenuated HFS (10 pulses at 40 Hz) for the different experimental groups (one-way ANOVA: *F*_(4,27)_ = 15.84, ***p < 0.001, followed by *post hoc* Dunnett’s test, ***p < 0.001). Stress/control data taken from Figures 2C,D is replotted in **(A–D)** for comparison.

LTP in the central nervous system often depends on NMDAR activation (Nicoll and Malenka, 1999; Morris, 2013; Volianskis et al., 2015). Subicular burst-spiking neurons are also known to express NMDAR-dependent LTP (Wozny et al., 2008b). In the presence of the NMDAR antagonist AP5, HFS failed to induce LTP (0.92 ± 0.09, *n* = 6/4, paired Student’s *t*-test; *p* = 0.38; Figure 4A). This suggests that the enhanced late-onset LTP after acute stress is NMDAR-dependent.

Dopamine is released in response to relevant stimuli, even aversive ones like foot shocks (Valenti et al., 2011). In addition, activation of D1/D5 dopamine receptors (D1/D5R) facilitates LTP in subicular burst-spiking neurons (Roggenhofer et al., 2010; Bartsch et al., 2015). Therefore, we tested whether D1/D5R need to be co-activated for the stress-enhanced LTP. After bath-application of the D1/D5R antagonist SCH23390, HFS resulted in a different time course of LTP with initially slightly reduced amplitudes (mixed model two-way ANOVA: interaction of “time” and “group”: *F*_(29,348)_ = 4.07, *p* < 0.0001; factor “time”: *F*_(29,348)_ = 16.01, *p* < 0.0001; factor “group”: *F*_(1,348)_ = 1.49, *p* = 0.25; Figure 4B). Yet, application of the D1/D5R antagonist SCH23390 failed to block subicular late-onset LTP in stressed rats (1.90 ± 0.17, *n* = 6/4, paired Student’s *t*-test, *p* < 0.01; Figure 4B). We conclude that the stress-enhanced late-onset LTP is D1/D5R-independent.

Rapid release of norepinephrine in the brain *via* activation of the locus coeruleus is a core neuroendocrine response to stressful stimuli. Acting *via* β-AR, norepinephrine can modulate hippocampal synaptic plasticity (Winder et al., 1999; Gelinas et al., 2008; Connor et al., 2012; Qian et al., 2012). Interestingly, subicular burst-spiking but not regular-spiking neurons were reported to express a β-AR-dependent form of LTP (Wójtowicz et al., 2010). Therefore, we tested a possible involvement of β-AR. Indeed, in the presence of the β-AR antagonist propranolol, late-onset LTP was blocked in stressed rats (1.00 ± 0.14, *n* = 6/2, paired Student’s *t*-test, *p* = 0.95; Figure 4C). Thus, the stress-enhanced subicular late-onset LTP is β-AR-dependent. A summary of changes in synaptic strength in the reported experiments is shown in Figure 4D.

## Discussion

The present findings confirm and extend previous studies on the stress-induced facilitating effect of norepinephrine and β-ARs in hippocampal LTP. We show that acute stress exposure in adult rats enhances LTP at ventral CA1—subiculum synapses tested *ex vivo* 1 day later. Stress-exposed rats show a reduced threshold for a late-onset LTP that relies on the activation of β-AR and NMDAR.

Stress has previously been shown to influence hippocampal synaptic plasticity. Most frequently, stress was linked to reduced hippocampal LTP (reviewed in Shors et al., 1989; Shors and Dryver, 1994; Kim et al., 1996, 2006; Mesches et al., 1999; Pavlides et al., 2002; Hirata et al., 2008; and Howland and Wang, 2008). However, studies indicate that stress-associated changes in LTP are not uniform. Rather, multiple factors affect the direction of change in LTP (Joëls and Krugers, 2007). The most relevant among them in relation to our findings are brain region and cell type of investigation, type of stress and the time point after stressor exposure at which experiments are conducted.

Concerning the area investigated, it is striking that the effects of acute stressors on hippocampal plasticity can vary prominently along the dorsoventral axis. Maggio and Segal (2011) tested LTP induction in the hippocampal area CA1 in slices from young adult rats (p 60) 1 day after exposure to acute stress (forced swim test). In line with our results, LTP in the ventral hippocampus was increased in slices from stressed rats compared to slices of control rats while LTP in dorsal hippocampus was impaired in the stress group (Maggio and Segal, 2011). Our findings further complement previous studies by MacDougall and Howland (2013a,b) demonstrating that acute stress disrupts LTP in rat dorsal subiculum. Considering our results, we conclude that, like in the CA1 region, acute stress has diverging effects on subicular LTP depending on the dorsoventral axis. Adding up to the complexity, previous reports revealed different stress-induced effects on LTP depending on the hippocampal subfield studied. A stressor can impair LTP in the dorsal CA1 while having no effect or even enhance LTP in the dentate gyrus (Izaki and Arita, 1996; Gerges et al., 2001, 2003; Kavushansky et al., 2006). Therefore, our and previous results argue for region-specific patterns of stress-induced changes in hippocampal LTP.

The type of stress also has an impact on stress-induced changes in LTP. Uncontrollable stress has impaired LTP in different areas of the hippocampus in previous studies [Diamond and Rose, 1994; Shors and Dryver, 1994 (dentate gyrus); Kim et al., 1996 (CA1), reviewed in Shors et al., 1997; Kim et al., 2006 (CA1)]. Rats able to terminate or escape electroshocks did not show LTP impairment (CA1, Shors et al., 1989). The two-way active avoidance conditioning employed in the present study poses an inherently stressful yet controllable situation on the animal. Thus, our results are compatible with these previous findings. The duration of stressor exposure also needs to be considered. Chronic stress exposure inhibits LTP in the hippocampus and medial prefrontal cortex [McEwen, 2001 (dorsal DG); Kim et al., 2006 (CA1 and dentate gyrus); Cerqueira et al., 2007 (prefrontal cortex)]. In accordance with our results, acute or brief stress exposure has been shown to facilitate LTP induction in the hippocampal—medial prefrontal cortex pathway and the lateral habenula (Goto and Grace, 2006; Park et al., 2017).

Next, the time lag between the end of the stress protocol and the assessment of synaptic plasticity is crucial when comparing findings from different studies due to the temporal profile of the stress response. It has been suggested that exposure to stress leads to a systemic stress response with an initial LTP-promoting phase followed by a later phase that may be LTP-preventing (Joëls and Krugers, 2007). We observed the stress-induced enhancement of LTP 1 day after acute stress exposure. Single exposure to swim stress has also been shown to enhance LTP *ex vivo* in the ventral CA1 up to 24 h later (Maggio and Segal, 2007, 2011). Thus, our results are in line with previously reported LTP-promoting effects. Moreover, stress activates multiple brain areas that are often mutually connected. For example, *via* basolateral amygdala—hippocampal (dentate gyrus) crosstalk, stress might induce time-dependent biphasic changes in hippocampal LTP with an initial enhancing effect followed by a depressing effect (Akirav and Richter-Levin, 1999).

This leads straight into the mechanisms underlying the stress-induced enhancement of LTP reported here. We demonstrated that the reduced induction threshold for LTP in ventral subiculum burst-spiking cells after acute stress is NMDAR- and β-AR-dependent. Antagonizing either NMDAR or β-AR alone was sufficient to block the stress-induced LTP. Thus, co-activation of NMDAR and β-AR is necessary for this form of stress-induced late-onset LTP at CA1—ventral subiculum synapses. Although not all forms of LTP require the activation of NMDAR, NMDARs are known for their important role in the induction of LTP for the majority of synapses in the central nervous system (reviewed in Morris, 2013; and Volianskis et al., 2015). Specifically, in subicular burst-spiking cells, the induction of LTP relies on the activation of NMDAR as well as on a rise in presynaptic Ca^2 +^, elevated cyclic AMP (cAMP) levels and the activation of cAMP-dependent protein kinase (PKA; Wozny et al., 2008a, b; Aoto et al., 2013). β-ARs are positively coupled to the adenylate cyclase and increase intracellular cAMP levels leading to activation of PKA. In the hippocampus, β-ARs can enhance NMDAR function by modulation of channel gating and Ca^2+^ permeability (Raman et al., 1996; Murphy et al., 2014). Most likely, PKA-mediated phosphorylation of the NMDAR GluN2B subunit is crucially involved in the β-AR-dependent facilitation of LTP induction (Murphy et al., 2014; O’Dell et al., 2015). This finding is in line with previous studies showing that acting through β-ARs, norepinephrine facilitates hippocampal LTP and memory storage (Stanton and Sarvey, 1985; Booze et al., 1993; Villani and Johnston, 1993; Winder et al., 1999; Lin et al., 2003; Gelinas et al., 2008; Wójtowicz et al., 2010; Connor et al., 2012; Qian et al., 2012; Ul Haq et al., 2012, 2016).

The ventral subiculum receives a strong noradrenergic innervation from the locus coeruleus (Oleskevich et al., 1989; Schroeter et al., 2000) and has a high density of ARs (Duncan et al., 1991; Booze et al., 1993). Stressful stimuli, such as foot shocks, can strongly activate noradrenergic neurons of the locus coeruleus and thereby alter ventral subiculum activity (Chang et al., 2000; Valentino and Van Bockstaele, 2008; Lipski and Grace, 2013b). In fact, Lipski and Grace have already shown that footshock inhibited 13%, and activated 48% of ventral subicular neurons by activating noradrenergic inputs from the locus coeruleus (Lipski and Grace, 2013b). Electrical locus coeruleus stimulation mimicked this effect. While inhibition was mediated primarily by α2-AR, activation was mediated by β-AR (Lipski and Grace, 2013a). Extracellular recordings have shown that β-AR-activation modulates LTP at ventral CA1—subiculum synapses (Huang and Kandel, 2005). Chemical activation of AR by the β-adrenergic agonist isoprotenerol has been reported to induce LTP in burst-spiking but not regular-spiking ventral subicular pyramidal cells (Wójtowicz et al., 2010; Grosser et al., 2015). Associated with stress, prenatal stress switched the LTP-enhancing effect of norepinephrine from dorsal to ventral hippocampus (Grigoryan and Segal, 2013). Juvenile stress also facilitated ventral hippocampal LTP and went along with an increased sensitivity to the β-AR agonist isoproterenol and an increased expression of β-ARs in the ventral hippocampus (Grigoryan et al., 2015). Accordingly, we present here that also adult stress facilitates LTP β-AR-dependently in the ventral subiculum. However, the timing of the pharmacological manipulations relative to the stressor in our experiments does not allow to decipher the potentially β-AR-dependent mechanisms triggered at the time of stress from those involved at the time of *ex vivo* LTP recordings 24 h later. It is feasible that our stress protocol can activate β-AR in ventral subiculum. Notably, release of endogenous noradrenaline by electrical stimulation in brain slices has been reported (Baldessarini and Kopin, 1966; Thienprasert and Singer, 1993) and previous acute stress with expected β-AR activation may have primed subiculum to this neuromodulatory input promoting LTP induction 24 h later. Hence, future studies with *ex vivo* LTP recordings conducted immediately following the two-way shuttle box training session are needed.

The initial LTP-promoting phase after stress exposure is thought to rely on catecholamines, peptides and nongenomic corticosterone actions involving mineralocorticoid receptors, while later stages of stress-induced alterations are linked to genomic glucocorticoid receptor-mediated actions. Recently, a third temporal domain of the stress response with a more delayed mode of action has been suggested (Joëls and Baram, 2009). It can be activated by the classically fast acting monoaminergic and peptidergic stress mediators by regulation of transcription factors (Sabban and Kvetnanský, 2001). Even though, stressors like foot shocks can activate the ventral subiculum leading to an increase in dopamine neuron population activity shortly after stress exposure (Valenti et al., 2011), and chemical activation of D1/D5R facilitates LTP at CA1—subiculum synapses (Roggenhofer et al., 2010), the stress-induced LTP reported here does not rely on D1/D5R activation.

Considering the results from PPI and CV^2^ analysis, several induction/expression mechanisms of the stress-induced LTP are conceivable. A contributing postsynaptic mechanism might include the phosphorylation and surface expression of the AMPA receptor subunit GluA1 that is important for the induction and maintenance of early LTP (Malenka, 2003; Plant et al., 2006; Kessels and Malinow, 2009). Indeed, this can be enhanced by application of norepinephrine or β-AR agonists (Hu et al., 2007; Tenorio et al., 2010; Zhou et al., 2012; Maity et al., 2015). Accordingly, stress might have activated β-AR signaling in our study. Moreover, β-AR-dependent recruitment of additional synaptic release sites could alter CV^2^ without affecting PPI (Manabe et al., 1993). Furthermore, β-AR signaling could enhance presynaptic NMDAR function leading to an increased glutamate release during HFS. This could thereby lower the induction threshold for LTP resulting in a facilitated LTP. Indeed, synaptic plasticity can rely on the activation of pre-synaptic NMDAR in various brain regions (Casado et al., 2002; Humeau et al., 2003; Sjöström et al., 2003; Duguid and Smart, 2004). Actually, the existence of presynaptic NMDAR mediating glutamate release in the subiculum has been suggested (Stan et al., 2014). Albeit direct anatomical evidence of β-ARs in pre-synaptic terminals of glutamatergic CA1—subiculum synapses is still missing, transport of β-ARs from the soma of CA1 pyramidal cells (Hillman et al., 2005; Guo and Li, 2007) to presynaptic sites is possible. Interestingly, β-AR signaling *via* direct activation of the guanine nucleotide exchange protein Epac by cAMP is involved in LTP at cerebellar granule cell to Purkinje cell synapses (Martín et al., 2020). There, an increase in the number of synaptic vesicles primed for exocytosis accounts for the potentiation of neurotransmitter release driven by β-ARs.

Our observation of an enhanced subicular LTP after stressful two-way active avoidance conditioning can be considered as a form of metaplasticity. Metaplasticity is an important mode of plasticity regulation and defined as a lasting modification in neuronal state following activation which impacts on the duration, magnitude or direction of future synaptic plasticity (Abraham and Bear, 1996; Hulme et al., 2013). Related to stress, metaplasticity might extend the time course of associativity of events, thereby preparing the individual for subsequent learning (Hulme et al., 2013). Even though, our data do not unequivocally prove a causal link between the enhanced LTP and a coping response, the subicular LTP facilitation was not due to mere learning during the behavioral paradigm as we found no correlation between LTP magnitude and rats’ performance during the task. However, all rats were able to learn the avoidance task under stressful conditions suggesting that LTP facilitation might indeed correlate with the stressful component of the operant learning task. Indeed, previous studies support the idea that stress-inhibitory influences of glutamatergic projections from ventral subiculum are exerted *via* a GABAergic relay in the anterior bed nucleus of stria terminalis (BNST) projecting to the paraventricular hypothalamic nucleus (Liu and Liang, 2009; Radley and Sawchenko, 2011). Furthermore, several studies have confirmed that stress induces norepinephrine release in BNST (Pacak et al., 1995; Cecchi et al., 2002; Pardon et al., 2002; Schmidt et al., 2019). Interestingly, Liu and Liang (2009) provide evidence that ventral subiculum can modulate memory formation in an inhibitory avoidance task *via* activation of glutamatergic and noradrenergic fibers innervating the BNST (Liu and Liang, 2009). Noradrenergic brainstem terminals and subicular glutamatergic afferents interact in the anterior BNST. By activating α2-AR, norepinephrine conveys tonic inhibitory control of glutamate release and glutamatergic postsynaptic activity in anterior BNST. This decrease in glutamatergic transmission may in return regulate GABAergic projections to the paraventricular hypothalamic nucleus arising from BNST (reviewed in Forray and Gysling, 2004). Ventral subiculum output and anterior BNST may thus process the stressful component of a learning task governed by noradrenergic neuromodulation.

Given the pivotal role of the subiculum as the critical hub in hippocampal information transfer to other brain regions and its role as a key regulator of the stress response, the enhanced LTP might therefore be behaviorally relevant and contribute to the animals’ stress adaptation.

## Data Availability Statement

The raw data supporting the conclusions of this article will be made available by the authors, without undue reservation.

## Ethics Statement

The animal study was reviewed and approved by Landesamt für Gesundheit und Soziales Berlin.

## Author Contributions

JCB, MC, UH and JB were responsible for conception of the study, design of the experiments and interpretation of the data. JCB, MC and DG acquired the data. JCB and MC analyzed the data. JCB and MC wrote the manuscript with inputs from JB. All authors contributed to the article and approved the submitted version.

## Conflict of Interest

The authors declare that the research was conducted in the absence of any commercial or financial relationships that could be construed as a potential conflict of interest.
